# *Helicobacter pylori* Seropositivity and Stool Antigen in Patients With Hyperemesis Gravidarum

**DOI:** 10.1155/IDOG/2006/73073

**Published:** 2006

**Authors:** R. Sinan Karadeniz, Ozlem Ozdegirmenci, M. Metin Altay, Ayse Solaroglu, Serdar Dilbaz, Nedret Hızel, Ali Haberal

**Affiliations:** Turkish Ministry of Health, Ankara Etlik Maternity and Women's Health Teaching Hospital, TR-06010 Etlik, Ankara, Turkey

## Abstract

The objective of this paper is to investigate whether *Helicobacter pylori* is an etiologic factor in hyperemesis gravidarum. Thirty one patients with hyperemesis gravidarum and twenty nine pregnant controls without hyperemesis gravidarum were included in this prospective study. All pregnant women were examined both for *Helicobacter pylori* serum immunoglobulin G antibodies (HpIgG Ab), showing chronic infection, and *Helicobacter pylori* stool antigens (HpSA), showing active gastrointestinal colonization. Chi-square and Student *t* tests were used accordingly for statistical analysis. *Helicobacter pylori* seropositivity was 67.7% in the patients with hyperemesis gravidarum and 79.3% in the control group (χ^2^ = 1.02, *P* = .31). HpSA was detected in 22.6% of patients with hyperemesis gravidarum, whereas 6.9% of patients in the control group. The difference was not statistically significant (χ^2^ = 2.89, *P* = .08). In this study, no relation was found between *Helicobacter pylori* and hyperemesis gravidarum. The low social status of women in both groups could be one of the reasons for the high prevalence of Hp infection.

## INTRODUCTION

Hyperemesis gravidarum (HG) is a common problem for an obstetrician. Nausea and vomiting of pregnancy, commonly known as “morning sickness,” affects approximately 80% of pregnant women and is generally a mild, self-limited condition that may be controlled with conservative measures [1]. A small percentage, 1–2%, of pregnant women have a more severe course, being HG. It is defined as vomiting in pregnancy which is pernicious to produce weight loss, dehydratation, acidosis from starvation, alkalosis from loss of hydrochloric acid, and hypokalemia [2]. All these symptoms are not absolutely necessary for the diagnosis. Mild to moderate ketonuria may be seen in urine analysis [3]. The typical onset is between 4 and 8 weeks' gestation, continuing until 14–16 weeks of gestation [4]. Although several theories have been proposed, the exact cause remains unclear. Several recent researches have implicated the *Helicobacter pylori* (Hp) as one possible cause [4–7], whereas there were also few recent studies that could not determine any relation between Hp and HG [8, 9]. So, the role of Hp in HG is controversial.

The purpose of this study is to investigate the possible association between Hp infection and HG in a group of Turkish pregnant women in first trimester.

## MATERIALS AND METHODS

Thirty one (51.7%) subjects and 29 (48.3%) controls were enrolled the study. Subjects were between 5 to 15 weeks' gestation and met the following criteria for hyperemesis gravidarum: severe vomiting (more than 3 times a day), weight loss (more than 5% of body weight), and ketonuria. Patients with known thyroid disease, multiple gestation, gestational trophoblastic disease, psychological and gastrointestinal disorders were excluded. Approval was obtained from the medical ethical committee and informed consents were obtained. Both groups were comparable for age, parity, and ultrasonographical age. Twenty nine pregnant women without symptoms of nausea and vomiting were involved the study as control group. Demographic data of both groups were recorded. Gestational age was determined using the first date of last menstrual period and confirmed by ultrasonography. The participants eligible for the study had been informed about the study before blood samples and stool specimens were collected.

### Determination of H pylori IgG Antibody

Samples were obtained by venipuncture and centrifuged at 3000 rpm for 10 minutes. Serum specimens were stored at −30°C until analysis. *H pylori* IgG antibody (HpIgG Ab) was measured using enzyme-linked immunosorbent assay (ELISA) kits (Virotech). Results were evaluated by BioTek ELx 800 ELISA reader. Test results were reported as positive, negative, and equivocal. The threshold value for a “positive” result was accepted as ≥ 1.00 and ≤ .90 as a negative result. Values between .91–.99 were interpreted as equivocal.

### Determination of H pylori Stool Antigen

Stool samples from each patient were collected into clean cups and stored at −30°C until analysis. All samples were tested for *H pylori* stool antigen (HpSA) using HpSA enzyme-linked immunosorbent assay (Diagnostic BioProbes srl, Milano, Italy) according to the manufacturers instructions. The cutoff value for a positive result was considered as ≥ .298 at optical density of 450 nm and < .298 as a negative result.

### Statistical analysis

Statistical analysis was performed by using SPSS 10.0 for Windows (SPSS Inc, Chicago, Ill, USA) statistical software. Comparison of serologic status of study and control groups for HpIgG Ab and HpSA was assessed by chi-square test. Descriptive statistics were shown as arithmetic mean ± standard deviation (SD). Student *t* test was used for comparing demographical properties of groups. *P* value less than .05 was considered as statistically significant.

## RESULTS

The ages of the women in both groups ranged from 17 to 40 (mean 26.05 ± 5.29). The mean duration of hospitalization in the study group was 3.22 ± .66 days. Both of the groups were similar in respect to their age, parity, and ultrasonographic age (Table 1).

The prevalence of HpIgG Ab was 67.7% (21 of 31) in the patients with HG, and 79.3% (23 of 29) in controls (*P* = .31; χ^2^ = 1.02). Positive HpSA was detected in 22.6% (7 of 31) in the study group, and 6.9% (2 of 29) in the control group (*P* = .08; χ^2^ = 2.89). There was no statistically significant difference in study and control groups for HpIgG Ab and HpSA (Table 2). Although 67.7% of the patients with HG had seropositivity, only 22.6% of them had an active gastrointestinal colonization. All of the patients who had HpSA positive had positive IgG antibodies except one.

## DISCUSSION

*Helicobacter pylori*, since its first isolation in 1982 by Marshall, represents one of the most common and medically prominent infections worldwide [10]. Infection with this microaerobic, gram-negative bacterium has been established as an etiologic factor in the development of peptic ulcer disease and has been associated with the development of gastric neoplasia [11]. *Helicobacter pylori* and its association with multiple gastroduodenal diseases have emphasized the importance of diagnosis of symptomatic individuals. The problems in diagnosis are more complicated during pregnancy since HG can mask an active Hp infection or HG may be severe by superimposed Hp infection.

The causative relation between Hp and some other diseases, beyond HG such as atherosclerosis, has been reported recently. Results of the studies suggest that gastrointestinal colonization of Hp increases atherosclerosis, formation of atheroma, and ischemic heart disease [12–15]. Likewise, the relation between Hp and HG has been studied in recent years [4, 5, 16]. Although their findings suggested a positive association between HG and Hp seropositivity, some recent studies could not find such association [8, 9]. Thus, this is one of the controversial issues in obstetric care. It was obvious that further studies were needed by tests other than serologic ones, which were more sensitive and specific. To date, all the studies investigating a relation with Hp and HG were done by serologic tests which can only be able to show chronic infections of Hp. Current study was planned for investigating the association between Hp and HG by using both serologic and stool antigen tests, the most recently developed test for *H pylori* in which the presence of the bacterium can be diagnosed with a sample of stool.

Currently, several diagnostic strategies are available for Hp infection. Serologic testing is a primary screening approach for evaluation of Hp status. The test shows IgG status of patients infected with Hp. In one study, seroconversion to IgG was demonstrated between 22 and 33 days after active infection [17]. Although serologic testing had the lowest cost per correct diagnosis, accuracy was lower than stool antigen testing [18]. The HpSA-ELISA test determines the colonization of *H pylori* in gastrointestinal tract by detecting Hp specific antigens in stool. Direct fecal antigen detection of Hp has been approved by the US Food and Drug Administration for diagnosis and followup testing [11]. The sensitivity and specificity of the HpSA test was found to be over 90% in patients with gastroduodenal ulcer diagnosed by gastric biopsy with rapid Hp urease test positivity. Vaira et al found the HpSA test 94.1% sensitive and 91.8% specific [19].

In this study, we were unable to confirm the reported association between Hp and HG by both diagnostic methods. Frigo et al reported that 90.5% of women with HG were seropositive for Hp as compared to 46.5% of controls [4]. Another study from Turkey with HG found that 92% were seropositive for Hp as compared to 45% of controls [5]. Although these two European studies strongly suggested that HG may be associated with Hp infection, two recent studies found no association between HG and Hp seropositivity, one conducted in two US populations with disparate Hp seropositivity [8] and the other by Berker et al from Turkey [9].

In our study, seropositivity is high in both study and control groups, 67.7% and 79.3%, respectively. One possible explanation to our result is the low socioeconomic level of our patient population. Numerous epidemiologic studies have shown that a major risk factor for Hp acquisition is low socioeconomic level in both developing and developed countries [8, 20]. Our findings support this association. Although serologic testing is the most common noninvasive diagnostic method for Hp and is relatively inexpensive and convenient, in our opinion a test that shows an active gastrointestinal colonization will be more appropriate in diagnosis of patients with HG.

In summary, we were unable to find an association between Hp and HG. The low social status of women in both groups could be one of the reasons for the high prevalence of Hp infection.

## Figures and Tables

**Table 1:** Demographic characteristics.

Characteristics	Hyperemesis (*n* = 31)	Control (*n* = 29)	*P*

Age	25.83 ± 4.68	26.27 ± 5.95	NS
Gravidity	1.83 ± .89	2.86 ± 1.40	.001
Parity	.64 ± .79	1.17 ± 1.03	NS
Gestational age (weeks)	9.63 ± 2.39	11.80 ± 2.70	.002
Ultrasonographic gestational age (weeks)	8.96 ± 2.06	9.62 ± 2.43	NS

**Table 2:** *Helicobacter pylori* seropositivity and fecal antigen status in hyperemesis gravidarum.

Test	Hyperemesis		Control		χ^2^	*P*
	Positive	Negative	Positive	Negative		

HpIgG	21 (67.7%)	10 (32.3%)	23 (79.3%)	6 (20.7%)	1.02	.31
HpSA	7 (22.6%)	24 (77.4%)	2 (6.9%)	27 (93.1%)	2.89	.08
